# Clinical outcome and outcome prediction of octogenarians with acute basilar artery occlusion and endovascular stroke treatment compared to younger patients

**DOI:** 10.3389/fneur.2023.1266105

**Published:** 2023-09-29

**Authors:** Charlotte Sabine Weyland, Matthias Anthony Mutke, Amanda Zimmermann-Miotk, Niclas Schmitt, Min Chen, Silvia Schönenberger, Markus Möhlenbruch, Martin Bendszus, Jessica Jesser

**Affiliations:** ^1^Department of Neuroradiology, Heidelberg University Hospital, Heidelberg, Baden-Wuürttemberg, Germany; ^2^Department of Neuroradiology, RWTH Aachen University, Aachen, Germany; ^3^Department of Neuroradiology, University Hospital of Basel, Basel, Switzerland; ^4^Department of Neurology, Heidelberg University Hospital, Heidelberg, Baden-Wuürttemberg, Germany

**Keywords:** thrombectomy, octogenarians, caucasian, basilar artery occlusion, outcome prediction, age, mortality, favorable outcome

## Abstract

**Background and aims:**

Octogenarians are underrepresented in recently published studies that showed the benefit of endovascular stroke treatment (EST) for patients with acute basilar artery occlusion (BAO). We aimed to compare the clinical outcome of octogenarians with BAO and EST compared to younger patients (YPs) and identify independent outcome predictors.

**Methods:**

This is a retrospective, single-center analysis of patients treated for BAO with EST from January 2013 until June 2021 in a tertiary stroke center. Octogenarians (80–89 years) were compared to YPs. A study endpoint was a favorable clinical outcome as per the modified Rankin Scale (mRS 0–3), 90 days after stroke onset. The study groups were compared using univariate analysis, and a multivariable logistic regression analysis was performed to define independent predictors for favorable and unfavorable (mRS 5–6) clinical outcomes.

**Results:**

In this study cohort, 74/191 (38.7%) octogenarians had a higher pre-stroke mRS [median, interquartile range (IQR): 2, 1–3 octogenarians vs. 0, 0–1 YP, *p* < 0.001] and a comparable National Institutes of Health Stroke Scale (NIHSS) before EST (median, IQR: 21, 10–38 vs. 20, 8–35 in YP, *p* = 0.487). They showed a comparable rate of favorable outcome (mRS 0–3, 90 days, 23.0 vs. 25.6% in YP, *p* = 0.725), but were less often functionally independent (mRS 0–2: 10.8% in octogenarians vs. 23.0% in YP, *p* = 0.049). The rate of unfavorable clinical outcome was comparable (mRS 5–6, *n* = 40, 54.1% in octogenarians vs. *n* = 64, 54.7% in YP, *p* = 0.831). A baseline NIHSS was an independent predictor for clinical outcome in YPs [e.g., for unfavorable clinical outcome: odds ratio (OR) 1.061, confidence interval (CI) 1.027–1.098, *p* = 0.005] and for favorable clinical outcome in octogenarians. Pre-stroke mRS predicted favorable outcomes in octogenarians (OR 0.54, CI 0.30–0.90, *p* = 0.0291), while age predicted unfavorable outcomes in YPs (OR 1.045, CI 1.011–1.086, *p* = 0.0137).

**Conclusion:**

Octogenarians with acute BAO eligible for EST are as likely to achieve a favorable outcome as YPs, and the rate of death or severe disability is comparable. The admission NIHSS is an independent predictor for favorable and unfavorable outcomes in YP and for favorable outcomes in octogenarians. In this study cohort, pre-stroke mRS predicted favorable outcomes in octogenarians while age predicted an unfavorable outcome in YPs.

## Introduction

Endovascular stroke treatment (EST) is an established first-line therapy for acute ischemic stroke and large-vessel occlusion (LVO) in the anterior circulation. There has been persistent doubt about the benefit of EST for basilar artery occlusions (BAO), which account for approximately 10% of all intracranial target vessel occlusions. After earlier studies could not prove the superiority of EST compared to the best medical treatment alone for BAOs (1–3), two recently published studies clearly showed the benefit of EST (4–6). Of these two studies, one excluded patients older than 80 years at the time of symptom onset in the BAOCHE trial (Trial of Thrombectomy 6–24 h after Stroke Due to Basilar-Artery Occlusion), the other included only 37 patients older than 80 years at symptom onset in the ATTENTION trial (Trial of Endovascular Treatment of Acute Basilar-Artery Occlusion), and both (BAOCHE and ATTENTION trials) were conducted in an exclusively Chinese population. This leaves a lack of knowledge concerning the outcome of octogenarians after EST for BAO in general, but especially in ethnicities other than Chinese. Further prospective, randomized controlled studies on EST for BAO will not be warranted after the ATTENTION and BAOCHE trials managed to show the superiority of EST compared to the best medical treatment. However, EST for BAO was performed for years before the recent trial publications, despite the missing evidence for endovascular stroke therapy, so a retrospective evaluation of the clinical outcome for octogenarians seems appropriate. Furthermore, when comparing BAO with anterior circulation LVOs, the improved outcome after EST was shown to be comparable, and the intervention was shown to be equally safe (7, 8). Ischemic stroke has a known age-dependent prevalence with a higher incidence in older age groups (9). For patients with anterior circulation stroke and EST, the knowledge gap concerning clinical outcomes after EST has already been closed, showing that octogenarians and even non-agenarians can still profit from EST (10, 11). In elderly Chinese patients from the Acute Basilar Artery Occlusion Study (BASILAR), Luo et al. showed that the outcome after EST was worse in patients older than 75 years but still more effective and safer than conservative treatment (12). In this study, we aimed to determine the clinical outcome of octogenarians presenting with BAO in comparison with younger patients eligible for EST in a Western-European population and to define independent predictors for the patients' clinical outcomes.

## Methods

The study protocol was approved by the local ethics committee, and patient informed consent was waived due to the retrospective study design (S-247/2009). This is a retrospective study based on a single-center stroke database of a tertiary stroke hospital with external patient referrals for EST from up to 10 hospitals during the study period. The institutional intention-to-treat stroke database was screened for patients who presented with a posterior circulation ischemic stroke due to a BAO and were treated with EST between January 2013 and June 2021. Stroke etiology was classified according to the TOAST classification: large-artery atherosclerosis, cardiac embolism, other known causes (e.g., dissection), embolic stroke of unknown source (ESUS), or unknown cause. The extent of the preinterventional ischemic lesion either on CT or MR imaging was rated by two neurointerventionalists by assessing the posterior circulation—Alberta Stroke Program Early CT Score (pcASPECTS) (13). The pcASPECTS is a predictor for functional outcome when transferred to diffusion-weighted MRI for ischemic lesions in the posterior circulation (14). Two trained neuroradiologists (each with 5 years of experience) determined the pcASPECTS in admission and follow-up imaging. Due to changing stroke imaging protocols during the assessment period of this study and varying clinical settings, CT and MR imaging were both evaluated depending on availability. The extent of final recanalization was rated in accordance with the modified Treatment In Cerebral Ischemia (mTICI) score by two neurointerventionalists (8 and 2 years of neurointerventional experience). A consensus reading was performed for the above-mentioned parameters (pcASPECTS and final recanalization mTICI). Complete reperfusion of the basilar artery as the target vessel (comparable to Arterial Occlusive Lesion classification 3) or failed recanalization with remaining occlusion of the target vessel (Arterial Occlusive Lesion classification 0–2) was interpreted separately by two neurointerventionalists.

### Performance of EST for LVO in the posterior circulation

The decision for EST was made by consensus between the neurologist and neurointerventionalist after initial stroke imaging with CT or MRI. Intravenous thrombolysis was administered according to national and international guidelines. The choice of the sedation mode in the complete study cohort was made according to the patient's compliance, severity of the stroke syndrome, and level of consciousness. In the standard approach for EST, a transfemoral access is performed, followed by placing a guide catheter in the subclavian artery (7F/80 cm Flexor Shuttle, Cook Medical, Bloomington, IN, USA). Subsequently, a distal access catheter is introduced to the vertebral artery. The material mostly used in this cohort is a 5F-Neuron intermediate catheter (2013–2014; Penumbra, Alameda, USA) or a 5F/6F-Sofia intermediate catheter (2013 until today; Microvention, Aliso Viejo, USA). All modern stent-retriever models are available in our facility with Solitaire (Medtronic), Trevo (Stryker, Kalamazoo, USA), and pREset (Phenox, Bochum, Germany) used most commonly for the posterior circulation. The first-line approach (performing contact aspiration or stent-retriever-thrombectomy in combination with continuous distal aspiration using a distal aspiration catheter) as well as the choice of material used for EST was at the discretion of the treating neurointerventionalist.

### Inclusion and exclusion criteria

**Figure 1:**
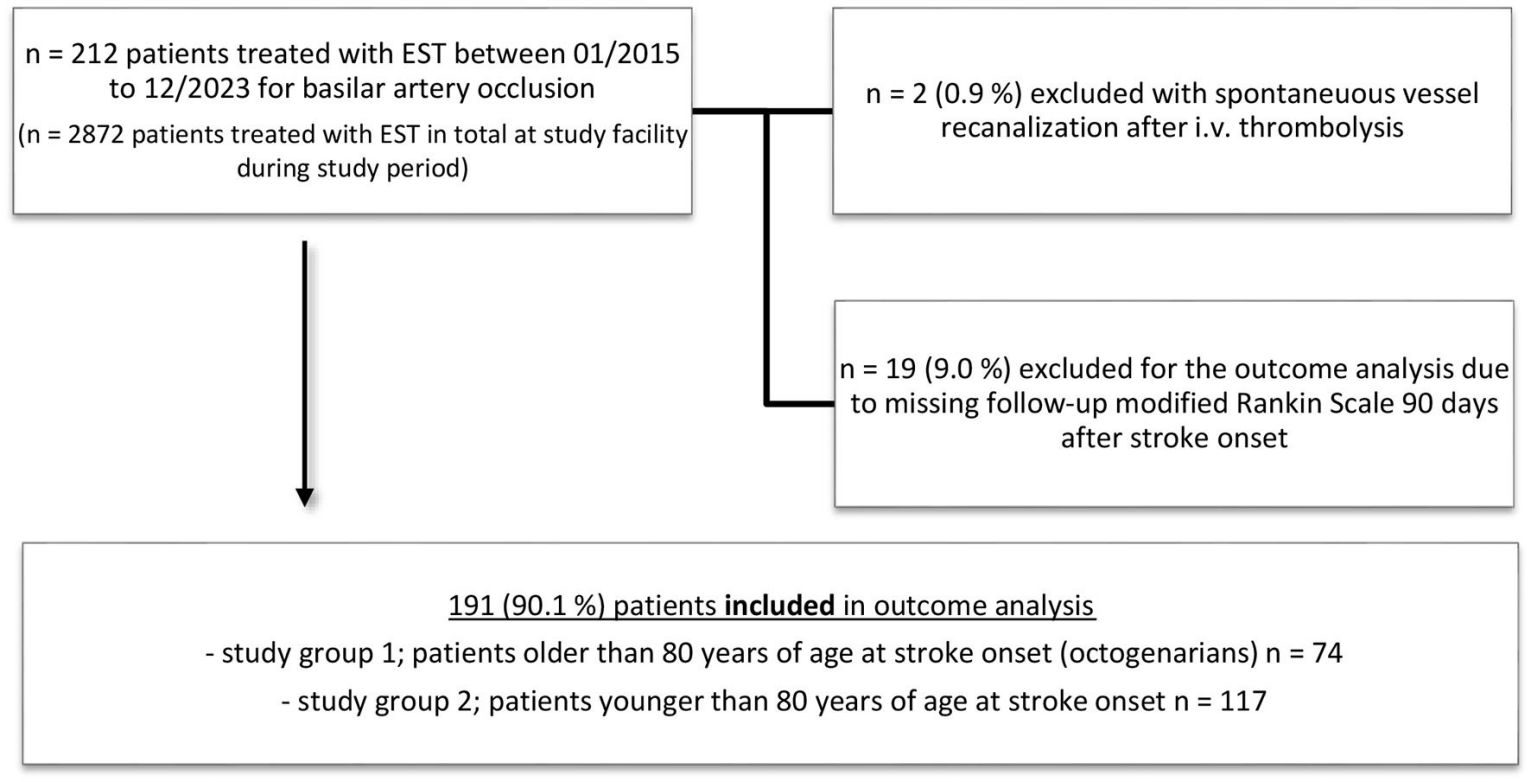
Patient selection and study groups for outcome comparison of octogenarians and younger patients with basilar artery occlusion treated with endovascular stroke treatment (EST).

All patients treated with EST of the posterior circulation involving the basilar artery on pre-interventional imaging were assessed for this study, i.e., 212 patients. In all, 111 patients were included based on CT imaging (non-contrast CT evaluation for pcASPECTS in all patients). Notably, 101 patients were included based on MR imaging (pcASPECTS evaluated with DWI available for all patients). Two patients with spontaneous recanalization of the target vessel occlusion after groin puncture (e.g., after i.v. thrombolysis) were excluded. For the outcome analysis, 19 patients with a missing modified Rankin Scale (mRS) 90 days after stroke onset were excluded. For patient selection and study groups, (see Figure 1). During the study period of 2,872 patients, a neurointervention was commenced with the intention of treating an acute ischemic stroke with a vessel occlusion in the anterior or posterior circulation.

### Study endpoints and multivariable regression analysis

The primary study endpoint was a favorable clinical outcome 90 days after stroke onset, defined as mRS 0–3. This primary study endpoint was chosen to define a positive clinical outcome because mRS 0–3 became most recently the dominant primary study endpoint in prospective, randomized posterior circulation EST trials (5, 6). Additionally, as good and unfavorable clinical outcomes according to mRS, we defined mRS 0–2 and mRS 5–6 as secondary outcome parameters. As a secondary analysis, a multivariable regression analysis involving clinical, imaging, and interventional parameters was performed for octogenarians and non-octogenarians to define independent predictors of favorable (mRS 0–3) and unfavorable (mRS 5–6) outcome—see Supplementary material for all parameters tested in the univariate regression analysis.

### Statistical analysis

Statistical analysis was performed with GraphPad PRISM (Version 9.0.1). Normality tests were performed, and statistical tests were chosen accordingly. Patients were classified into octogenarians and younger patients at the time of stroke onset.

Groups were compared for differences in stroke etiology, clinical outcome after 90 days, and thrombectomy technique using a Kruskal–Wallis test with Dunn's correction for multiple comparisons or the Mann–Whitney *U*-test when combining groups and comparing two groups only. Frequency of successful recanalization (defined as mTICI 2b, 2c, or 3), type of first thrombectomy attempt, and frequency of stenting across different occlusion groups were compared using a chi-squared test for multiple groups or Fisher's exact test for two groups in cases where groups were combined. All tests were performed on the basis of a two-sided *p*-value of less than 0.05, which was considered statistically significant.

## Results

In this study cohort, the octogenarians were more often women compared to the younger patients [men, *n* (%), 31 (42%) octogenarians vs. 76 (65%) younger, *p* = 0.027]. They also had higher posterior-circulation ASPECTS before endovascular stroke treatment [pcASPECTS, median (IQR), 10 (8–10) octogenarians vs. 9 (7–10) younger patients, *p* = 0.0137]. On admission, they had comparable neurological deficits [National Institutes of Health Stroke Scale (NIHSS), median (IQR), 21 (10–38) octogenarians vs. 20 (8–35) younger, *p* = 0.4867], and higher pre-stroke disability as per modified Rankin Scale [pre-stroke mRS, median (IQR), 2 (1–3) octogenarians vs. 0 (0–1) younger, *p* < 0.0001] (see Table 1). Octogenarians were more often diagnosed with atrial fibrillation before stroke onset [atrial fibrillation, *n* (%), 37 (50%) octogenarians vs. 37 (32%) younger, *p* = 0.0145], while other-stroke related comorbidities were comparable between study groups.

**Table 1:** Demographic, interventional, and imaging parameters of octogenarians and younger patients with acute ischemic stroke and basilar artery occlusion eligible for endovascular stroke treatment.

	**Octogenarians 80–89 years (*n* = 74)**	**Younger patients < 80 years (*n* = 117)**	***p*-value**
Age, median (IQR)	84 (82–86)	70 (59–75)	**< 0.0001**
Sex, male; *n* (%)	31 (42)	76 (65)	**0.0027**
Referred to stroke center from other hospital; *n* (%)	35 (47)	73 (62)	0.0706
Wake-up stroke; *n* (%)	31 (42)	43 (37)	0.5426
Posterior-circulation ASPECTS on imaging before EST, median (IQR)	10 (8–10)	9 (7–10)	**0.0137**
Time from symptom onset to recanalization, min (IQR)	434 (301–702)	467 (293–967)	0.4158
Time from symptom onset to groin puncture, median (IQR)	331 (209–576)	390 (221–817)	0.2138
i.v. thrombolysis; *n* (%)	33 (45)	51 (44)	>0.999
NIHSS admission, median (IQR)	21 (10–38)	20 (8–35)	0.4867
Pre-stroke mRS, median (IQR)	2 (1–3)	0 (0–1)	**< 0.0001**
**Comorbidities;** ***n*** **(%)**			
Diabetes mellitus type 2	21 (28)	21 (18)	0.1058
Arterial hypertension	67 (91)	90 (77)	0.0656
Coronary artery disease	28 (38)	29 (25)	0.0746
Dyslipidemia	27 (36)	33 (28)	0.3337
Atrial fibrillation	37 (50)	37 (32)	**0.0145**
**Interventional details during EST**			
Successful recanalization of basilar artery occlusion; *n* (%)	64 (86)	107 (91)	0.3338
Number of thrombectomy attempts; median (IQR)	1 (1–2)	1 (1–3)	0.6426
Intracranial stenting during EST	16 (22)	24 (21)	0.8571
Stent-retriever thrombectomy as first approach; *n* (%)	54 (73)	79 (68)	0.3162
Contact aspiration as first approach; *n* (%)	10 (14)	28 (24)	0.089
Direct intracranial stenting, no thrombectomy attempt; *n* (%)	4 (5)	5 (4)	0.970
Occlusion not reached; *n* (%)	5 (7)	3 (3)	0.257
Conscious sedation as sedation mode during EST; *n* (%)	30 (41)	46 (39)	0.891
**Intracranial hemorrhage in follow-up imaging**			
Intracranial hemorrhage in follow-up imaging, *n* (%)	14 (19)	17 (15)	0.652
Symptomatic intracranial hemorrhage in follow-up imaging, *n* (%)	5 (7)	6 (5)	0.821
**Stroke etiology;** ***n*** **(%)**			
Atherosclerosis	23 (31)	37 (32)	0.4218
Cardio-embolic	31 (42)	36 (31)	0.127
Other causes	1 (1)	4 (3)	0.393
Embolic stroke of unknown source	10 (14)	25 (21)	0.189
Unknown (work-up not completed)	9 (12)	15 (13)	0.928
Clinical outcome 90 days after stroke onset per modified Rankin Scale (mRS), n (%)			
Missing values: octogenarians = 7, non-octogenarians = 12			
Favorable outcome (mRS 0–3); *n* (%)	17 (23.0)	30 (25.2)	0.725
mRS 90 days after; median (IQR)	5 (2-6)	5 (3-6)	0.092
Difference between mRS 90 and pre-stroke mRS	4 (1-6)	3 (2-4)	**< 0.001**
Good outcome (mRS 0-2); *n* (%)	8 (10.8)	27 (23.0)	**0.0485**
Unfavorable outcome (mRS 5–6); *n* (%)	40 (54.1)	64 (54.7)	0.831
mRS 4; *n* (%)	17 (23.0)	13 (11.1)	**0.041**
Mortality (mRS 6); *n* (%)	34 (45.9)	55 (47.0)	0.643

ASPECTS, Alberta Stroke Program Early CT Score; EST, Endovascular Stroke Treatment; IQR, interquartile range; mRS, modified Rankin Scale; NIHSS, National Institutes of Health Stroke Scale; i.v., intravenous. Significant values are printed in bold.

The rate of conscious sedation as the sedation mode during EST was comparable between study groups (39% in octogenarians vs. 41% in younger patients, *p* = 0.891). Both study groups showed the same rate of successful target vessel (basilar artery) recanalization (see also Supplementary material for complete mTICI rates), a comparable rate of intracranial stenting during EST, and the first-line approach when comparing contact aspiration and stent-retriever thrombectomy did not differ (see Table 1). For octogenarians, the leading cause of stroke was cardioembolic with a significantly higher rate of cardioembolic stroke compared to younger patients [*n* (%), 31 (42%) octogenarians vs. 36 (31%) younger patients]. Younger patients showed more often an ESUS than octogenarians, while atherosclerosis was as likely as cardioembolic stroke as stroke etiology in younger patients (see Table 1).

**Figure 2:**
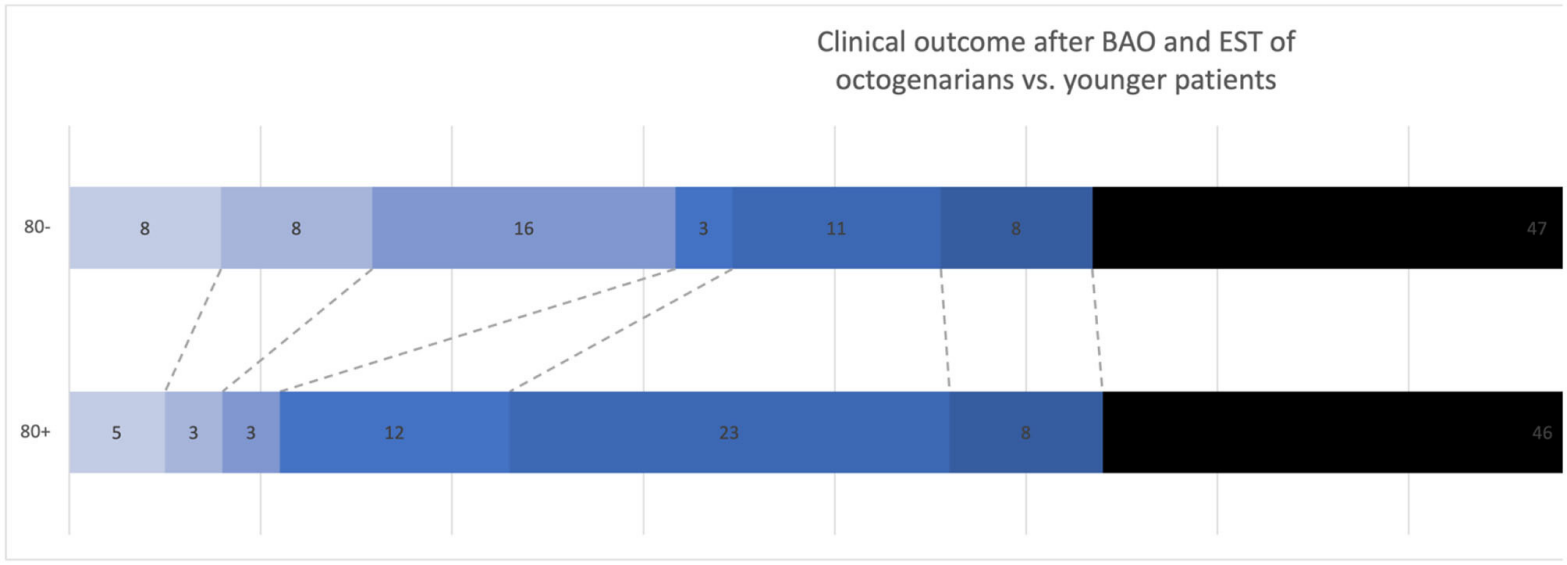
Group comparison of clinical outcome as per modified Rankin Scale 90 days after stroke onset of young patients (80–) vs. octogenarians (80+). BAO, Basilar Artery Occlusion; EST, Endovascular Stroke Treatment. Numbers in the bars indicate the percentage of patients in the mRS subgroup.

When comparing the outcome, younger patients were more often functionally independent 90 days after stroke onset [mRS 0–2, *n* (%), 8 (10.8%) octogenarians vs. 27 (23.0%) younger patients, *p* = 0.0485) but showed a comparable rate of favorable clinical outcome [mRS 0–3, *n* (%), 17 (23.0%) octogenarians vs. 30 (25.2%) younger patients, *p* = 0.725]. For unfavorable outcomes, comprising severe handicap or death 90 days after stroke onset, the two study groups showed no difference [mRS 5–6, *n* (%), 40 (54.1%) octogenarians vs. 64 (54.7%) younger patients, *p* = 0.831]; see Figure 2 for group comparison and Supplementary material for comparison of pre-stroke mRS to post-stroke mRS within the study groups.

Independent predictors for octogenarians regarding a favorable outcome (mRS 0–3, 90 days after stroke onset) were the NIHSS on admission [OR 0.92, confidence interval (CI) 0.86–0.97, *p* = 0.0019] and pre-stroke mRS (OR 0.54, CI 0.30–0.90, *p* = 0.0291). For an unfavorable outcome (mRS 5–6 after 90 days), a higher NIHSS on admission showed a trend for unfavorable outcomes (OR 1.04, CI 1.00–1.085, p = 0.0591) (see Table 2).

**Table 2:** Independent predictors of favorable clinical outcome (mRS 0–3) and unfavorable clinical outcome (mRS 5–6) in logistic regression analysis for octogenarians.

	**Favorable clinical outcome (mRS 0–3)**			**Unfavorable clinical outcome (mRS 5–6)**		
	**Odds ratio**	**Confidence interval**	***p*** **-value**	**Odds ratio**	**Confidence interval**	***p*** **-value**
NIHSS on admission	0.9192	0.86–0.97	**0.0019**	1.04	1.00–1.085	0.0591
Pre-stroke mRS	0.5423	0.30–0.90	**0.0291**	1.401	0.9141–2.220	0.1486
Age (years)	0.9001	0.7503–1.050	0.3445	0.9924	0.8717–1.132	0.7099

Significant values are printed in bold.

For younger patients, the NIHSS on admission was likewise an independent predictor for favorable outcome (OR 0.94, CI 0.90–0.97, *p* = 0.0002) and unfavorable outcome (OR 1.06, CI 1.027–1.098, *p* = 0.005) (see Table 3). Moreover, in younger patients, older age was predictive of an unfavorable outcome (OR 1.045, CI 1.011–1.086, *p* = 0.00137).

**Table 3:** Independent predictors of favorable clinical outcome (mRS 0–3) and unfavorable clinical outcome (mRS 5–6) in logistic regression analysis for younger patients.

	**Favorable clinical outcome (mRS 0–3)**			**Unfavorable clinical outcome (mRS 5–6)**		
	**Odds ratio**	**Confidence interval**	***p*** **-value**	**Odds ratio**	**Confidence interval**	***p*** **-value**
NIHSS on admission	0.935	0.90–0.97	**0.0002**	1.061	1.027–1.098	**0.005**
Pre-stroke mRS	0.7356	0.4638–1.107	0.2157	1.47	0.9898–2.284	0.0675
Age (years)	0.9577	0.9232–0.9891	0.073	1.045	1.011–1.086	**0.0137**

Significant values are printed in bold.

## Discussion

In this study of patients treated with EST for acute ischemic stroke due to BAO, octogenarians were as likely as younger patients to achieve a favorable outcome but less likely to be functionally independent 90 days after stroke treatment. This lack of functional independence (mRS 0–2) is connected to the octogenarians' higher pre-stroke mRS compared to younger patients. Octogenarians had the same risk of experiencing an unfavorable outcome with severe handicap or death (mRS 5–6) compared to younger patients.

Our results are comparable to studies referring to anterior circulation LVOs that showed that very old patients could still possibly benefit from EST (15, 16). Regarding Western-European study populations, this is the first study in the era of modern EST to investigate clinical outcomes for octogenarians after BAO and EST compared to younger patients. Vergouwen et al. (17) showed in 2012, for patients older than 75 years, that elderly patients can still profit from EST, although the mortality rate was higher for the elderly, which contradicts our study results. This study is also comparable to a Chinese population-based study (BASILAR) by Luo et al. (12) reporting that patients older than 75 years (mean age with IQR: 78, 76–81) at stroke onset still benefit from EST compared to the best medical treatment. Luo et al. also reported a higher mortality rate (54% in patients older than 75 years) compared to our study results (47% in octogenarians). Likewise, in the intervention arm of the BASILAR study, the admission NIHSS was also an independent predictor for clinical outcome, whereas pre-stroke mRS was not investigated in that study, which proved to be predictive of a favorable outcome in our study. Contrary to our study results, there was a higher mortality rate for older patients, but the rate of favorable outcomes was also comparable between age groups. Contrary to Luo et al., there were more female stroke patients among octogenarians in our study cohort. This might be due to the overall lower life expectancy in China (i.e., in 2022: 76.0 years in men and 81.3 years in women) compared to Germany (78.5 years in men and 83.5 years in women) (16). Further studies concerning sex differences in ischemic stroke treatment and outcome are warranted to define important sex-related differences in this disease in the elderly due to the higher life expectancy of women. Deb-Chatterji et al. (18) found that sex alone is not an independent predictor for ischemic stroke outcome but that the worse clinical outcome for women after acute stroke care is crucially confounded by the higher representation of women in the elderly group and the age-associated higher pre-stroke functional status.

Although posterior-circulation (pc) ASPECTS was lower in the younger group of our study by one point, it was not an independent predictor of a favorable or unfavorable outcome. Ouyang et al. (19) found a cutoff value of a preinterventional pcASPECTS of 8, grouping patients into pcASPECTS 0–7 vs. 8–10, to work best in differentiating a good vs. poor outcome. We suppose that the difference of one point with a median of 10 and 9, respectively, in the octogenarian and younger patient groups might not be large enough to distinguish favorable vs. unfavorable patient outcomes (18). Posterior fossa artifacts in CT imaging make pcASPECTS less reliable compared to ASPECTS for anterior circulation occlusions.

Luo et al. (12) found a higher rate of cardioembolic stroke in the elderly, which is in line with our study results. While atherosclerotic disease is overall more prevalent in posterior circulation stroke compared to anterior circulation stroke, the higher incidence of atrial fibrillation in older patients, especially in women, leads to a higher prevalence of cardioembolic stroke origin (20, 21). They did not report on the rate of ESUS as a stroke etiology, which proved to be higher in younger patients in our study cohort compared to octogenarians. ESUS has a known age-related distribution in stroke patients with a higher likelihood in younger patients (22). In our study, more octogenarian patients were diagnosed with atrial fibrillation, yet atrial fibrillation was not found to be an independent predictor of favorable or unfavorable outcomes, which might be related to the high number of patients showing atrial fibrillation in all subgroups. The negative impact of atrial fibrillation on rehabilitation potential and its association with infarct growth and hemorrhagic transformation in stroke have been shown in several studies. In acute BAO, atrial fibrillation was found to have no influence on the safety and efficacy of EST but was found to be associated with infarct recurrence within 1 year (23, 24). Approximately 50% of patients in our study (47 and 62%, respectively) were transferred from other hospitals for EST, which could contribute to the slightly longer time from symptom onset to recanalization compared to other studies. In the ATTENTION trial [median of stroke onset to revascularization was 6.9 h (IQR 5.0–8.8), whereas in our study it was 7.2 h (5.0–11.7) in octogenarians vs. 7.7 h (4.9–16.1) in younger patients, *p* = 0.416] (3). The rate of intravenous thrombolysis was comparable between study groups and higher compared to the thrombolysis rate of 31% in the intervention arm of ATTENTION. Systemic thrombolysis as bridging thrombolysis before EST did not differ between study groups and is still discussed but is currently maintained as beneficial compared to EST alone (23, 24).

The limitations of this study concern the single-center retrospective study design and the rather long study period. The rate of successful basilar artery recanalization did not increase in more recent years (see Supplementary material). The sedation mode during EST (conscious sedation or general anesthesia) can influence the study results. At the study facility, conscious sedation for posterior circulation EST was shown to be feasible for eligible patients (25). The rate of conscious sedation as a sedation mode as opposed to general anesthesia was comparable between the study groups. More research is warranted to assess the influence of anesthesia management in posterior circulation EST on patient outcomes. The distribution of stroke etiology can differ depending on the geographical background of the study population. A population's life expectancy also influences study results concerning elderly patients and acute ischemic stroke care. Due to the demographic change in Western populations, further research is still warranted to address the treatment benefit of EST in older populations.

## Conclusion

Octogenarians are as likely as younger patients to achieve a favorable outcome after BAO and EST and experience a comparable rate of severe handicap or death. The admission NIHSS is an independent predictor of favorable and unfavorable outcomes in younger patients and favorable outcomes in octogenarians. In this study cohort, pre-stroke mRS predicted favorable outcomes in octogenarians, while age predicted unfavorable outcomes in younger patients.

## Data availability statement

The raw data supporting the conclusions of this article will be made available by the authors, without undue reservation.

## Ethics statement

The studies involving humans were approved by the Ethikkommission Medizinische Fakultät Universität Heidelberg. The studies were conducted in accordance with the local legislation and institutional requirements. The Ethics Committee/Institutional Review Board waived the requirement of written informed consent for participation from the participants or the participants' legal guardians/next of kin because due to the retrospective study design.

## Author contributions

CW: Conceptualization, Data curation, Formal analysis, Investigation, Methodology, Supervision, Writing—original draft, review, and editing. MMu: Conceptualization, Data curation, Formal analysis, Investigation, Methodology, Writing—original draft, review, and editing. AZ-M: Data curation, Investigation, Writing—original draft. NS: Conceptualization, Data curation, Writing—review and editing. MC: Conceptualization, Data curation, Writing—original draft. SS: Data curation, Writing—original draft. MMö: Data curation, Project administration, Resources, Validation, Writing—review and editing. MB: Project administration, Resources, Validation, Writing—review and editing. JJ: Conceptualization, Data curation, Formal analysis, Investigation, Methodology, Validation, Writing—original draft, review, and editing.
