# Supplementary material for: Clinical outcome and outcome prediction of octogenarians with acute basilar artery occlusion and endovascular stroke treatment compared to younger patients
Source: Front Neurol. 2023 Sep 29;14:1266105. doi: 10.3389/fneur.2023.1266105 (PMC10570514; doi:10.3389/fneur.2023.1266105)
Supplement: Supplementary file 1 [file Data_Sheet_1.docx]

Supplementary Material

mTICI Score after endovascular stroke treatment

| mTici | 80 + | 80 - |
| --- | --- | --- |
| 0 | 6 | 4 |
| 1 | 1 | 1 |
| 2a | 3 | 5 |
| 2b | 13 | 37 |
| 2c | 7 | 12 |
| 3 | 40 | 58 |
| Missing data | 1 | 3 |
|  |  |  |
| All patients | 71 | 120 |
| all included | 70 | 117 |
| Successful recanalization (mTICI 2b-3) | 60 | 107 |
| Failed recanalization (mTICI 0-2a) | 23 | 47 |

mTICI = modified Treatment In Cerebral Infarction score

Successful and failed basilar artery occlusion recanalization over the years

| Year | Successful (mTici 2b-3) | Failed (mTICI 0-2a) | all | missing |
| --- | --- | --- | --- | --- |
| 2013 | 10 | 6 | 16 |  |
| 2014 | 13 | 2 | 16 | 2 |
| 2015 | 10 | 2 | 22 |  |
| 2016 | 19 | 1 | 20 |  |
| 2017 | 29 | 4 | 33 |  |
| 2018 | 21 | 8 | 30 | 1 |
| 2019 | 22 | 6 | 30 | 2 |
| 2020 | 12 | 1 | 13 |  |
| 2021 | 11 | 0 | 11 |  |

mTICI = modified Treatment In Cerebral Infarction score

BAO = basilar artery occlusion; successful recanalization = modified Treatment In Cerebral Infarction score 2b – 3

Complete mRS analysis according to study groups and pre-stroke/post-stroke mRS

| Pre-stroke mRS  (4 missing values) | 80+ | 80- |  | Post-stroke mRS 90d after stroke onset | 80+ | 80- |
| --- | --- | --- | --- | --- | --- | --- |
| 0 | 8 | 72 |  | 0 | 4 | 9 |
| 1 | 22 | 16 |  | 1 | 2 | 9 |
| 2 | 19 | 16 |  | 2 | 2 | 19 |
| 3 | 18 | 10 |  | 3 | 9 | 3 |
| 4 | 3 | 1 |  | 4 | 17 | 13 |
| 5 | 1 | 1 |  | 5 | 6 | 9 |
| 6 | 0 | 0 |  | 6 | 34 | 55 |

80+ = octogenarians, 80- = younger patients, mRS = modified RankinScale

Atrial fibrillation

37/117 (32%) of younger patients in our study cohort suffered from atrial fibrillation, 7 of them on NOAC as home medication, 4 using another anticoagulation medication.

37/74 (50%) of octogenarian patients in our study cohort suffered from atrial fibrillation, 11 of them on NOAC as home medication, 8 using another anticoagulation medication.

We have no information about the effectiveness or blood levels of the anticoagulation medication or the patient’s compliance.

Regression Analysis

Independent predictors of favorable clinical outcome (mRS 0-3) and unfavorable clinical outcome (mRS 5-6) in logistic regression analysis for octogenarians

|  | **Favorable clinical outcome (mRS 0-3)** | | | **Unfavorable** **clinical outcome (mRS 5-6)** | | |
| --- | --- | --- | --- | --- | --- | --- |
|  | Odds Ratio | Confidence interval | P-value | Odds Ratio | Confidence interval | P-value |
| **Successful recanalization** | 0.268 | 0.01 - 1.59 | 0.4363 | 0.119 | 0.006252 - 0.6938 | 0.1687 |
| **Number of thrombectomy maneuvers** | 0.9127 | 0.5875 – 1.322 | 0.6414 | 0.7929 | 0.5270 - 1.125 | 0.2725 |
| **Intracranial stenting** | 0.5707 | 0.1646 – 2.131 | 0.5408 | 0.9892 | 0.3022 - 3.375 | 0.9901 |
| **Age (years)** | 0.9001 | 0.7503 – 1.050 | 0.3445 | 0.9924 | 0.8717 - 1.132 | 0.7099 |
| **Sex** | 0.9052 | 0.3051 – 2.740 | 0.8785 | 0.713 | 0.2632 - 1.888 | 0.5642 |
| **Transfer - center from external clinic** | 0.7778 | 0.2593 – 2.325 | 0.6975 | 1.769 | 0.6624 - 4.823 | 0.3305 |
| **i.v. thrombolysis** | 0.6897 | 0.2298 – 2.059 | 0.5667 | 0.8951 | 0.3361 - 2.387 | 0.8495 |
| **NIHSS on admission** | 0.9192 | 0.8639 – 0.9675 | **0.0019** | 1.04 | 1.000 - 1.085 | *0.0591* |
| **Pre-stroke mRS** | 0.5423 | 0.3032 – 0.9039 | **0.0291** | 1.401 | 0.9141 - 2.220 | 0.1486 |
| **Diabetes** | 2.656 | 0.7452 – 12.62 | 0.2608 | 0.3194 | 0.09166 - 0.9727 | 0.1211 |
| **Arterial hypertension** | 0.4314 | 0.02200 – 2.791 | 0.6869 | 1.985 | 0.3938 - 14.62 | 0.6778 |
| **Coronary heart disease** | 2.217 | 0.6768 – 8.724 | 0.3018 | 1.296 | 0.4680 - 3.705 | 0.6855 |
| **Arrhythmia** | 1.534 | 0.5186 – 4.662 | 0.5062 | 0.4604 | 0.1685 - 1.220 | 0.1819 |
| **Dyslipidaemia** | 1.379 | 0.4547 – 4.524 | 0.6407 | 0.7237 | 0.2607 - 1.955 | 0.5951 |
| **Wake-up stroke** | 2.043 | 0.6597 – 7.162 | 0.3102 | 0.8785 | 0.3235 - 2.347 | 0.8282 |
| **pcASPECTS on first imaging (CT or MRI)** | 1.211 | 0.8231 – 1.938 | 0.338 | 0.7874 | 0.5343 - 1.108 | 0.1738 |
| **Time from symptom onset to final recanalization (in min)** | 0.9989 | 0.9968 - 1.000 | 0.5032 | 0.9999 | 0.9991 - 1.001 | 0.5309 |
| **Time from symptom onset to groin puncture (in min)** | 0.999 | 0.9967 - 1.000 | 0.5782 | 0.9998 | 0.9989 - 1.001 | 0.833 |

Independent predictors of favorable clinical outcome (mRS 0-3) and unfavorable clinical outcome (mRS 5-6) in logistic regression analysis for non-octogenarians

|  | **Favorable clinical outcome (mRS 0-3)** | | | **Unfavorable** **clinical outcome (mRS 5-6)** | | |
| --- | --- | --- | --- | --- | --- | --- |
|  | Odds Ratio | Confidence interval | P-value | Odds Ratio | Confidence interval | P-value |
| **Successful recanalization** | 0.246 | 0.01290 - 1.462 | 0.5289 | 5.943 | 1.004 - 113.3 | 0.1014 |
| **Number of thrombectomy maneuvers** | 0.84 | 0.6304 - 1.072 | 0.2598 | 1.177 | 0.9355 - 1.531 | 0.1898 |
| **Intracranial stenting** | 1.007 | 0.3886 - 2.759 | 0.9921 | 0.6361 | 0.2482 - 1.613 | 0.3391 |
| **Age (years)** | 0.9577 | 0.9232 - 0.9891 | *0.073* | 1.045 | 1.011 - 1.086 | **0.0137** |
| **Sex** | 1.816 | 0.7977 - 4.153 | 0.2342 | 0.6024 | 0.2694 - 1.335 | 0.2129 |
| **Transfer - center from external clinic** | 1.659 | 1.147 - 2.428 | >0.9999 | 0.967 | 0.4394 - 2.139 | 0.9334 |
| **i.v. thrombolysis** | 0.9314 | 0.4117 - 2.113 | 0.8831 | 0.8889 | 0.4033 - 1.949 | 0.7687 |
| **NIHSS on admission** | 0.9348 | 0.9002 - 0.9673 | **0.0002** | 1.061 | 1.027 - 1.098 | **0.0005** |
| **Pre-stroke mRS** | 0.7356 | 0.4638 - 1.107 | 0.2157 | 1.47 | 0.9898 - 2.284 | *0.0675* |
| **Diabetes** | 1.253 | 0.4502 - 3.853 | 0.7753 | 0.6694 | 0.2310 - 1.809 | 0.4387 |
| **Arterial hypertension** | 1.038 | 0.3555 - 2.841 | 0.9606 | 1.527 | 0.5647 - 4.429 | 0.4144 |
| **Coronary heart disease** | 1.778 | 0.6888 - 5.021 | 0.376 | 0.5429 | 0.2083 - 1.340 | 0.1943 |
| **Arrhythmia** | 1.025 | 0.4360 - 2.472 | 0.963 | 0.9569 | 0.4144 - 2.185 | 0.9169 |
| **Dyslipidaemia** | 1.096 | 0.4513 - 2.770 | 0.8744 | 0.7946 | 0.3292 - 1.869 | 0.6015 |
| **Wake-up stroke** | 2.256 | 0.9503 - 5.724 | 0.1342 | 0.4615 | 0.1958 - 1.046 | *0.0689* |
| **pcASPECTS on first imaging (CT or MRI)** | 1.193 | 0.9431 - 1.543 | 0.1856 | 0.8263 | 0.6480 - 1.035 | 0.1074 |
| **Time from symptom onset to final recanalization (in min)** | 1 | 0.9998 - 1.000 | 0.7706 | 1 | 0.9997 - 1.000 | 0.7168 |
| **Time from symptom onset to groin puncture (in min)** | 1 | 0.9998 - 1.000 | 0.9709 | 0.9999 | 0.9997 - 1.000 | 0.678 |
